# Identification of genes affecting alginate biosynthesis in *Pseudomonas fluorescens* by screening a transposon insertion library

**DOI:** 10.1186/s12864-016-3467-7

**Published:** 2017-01-03

**Authors:** Helga Ertesvåg, Håvard Sletta, Mona Senneset, Yi-Qian Sun, Geir Klinkenberg, Therese Aursand Konradsen, Trond E. Ellingsen, Svein Valla

**Affiliations:** 1 Department of Biotechnology, NTNU-Norwegian University of Science and Technology, Trondheim, Norway; 2 SINTEF Materials and Chemistry, Trondheim, Norway

**Keywords:** *Pseudomonas fluorescens*, Alginate biosynthesis, Transposon mutants, Fructose 6-phosphate, Purine, Tryptophan, Peptidoglycan recycling

## Abstract

**Background:**

Polysaccharides often are necessary components of bacterial biofilms and capsules. Production of these biopolymers constitutes a drain on key components in the central carbon metabolism, but so far little is known concerning if and how the cells divide their resources between cell growth and production of exopolysaccharides. Alginate is an industrially important linear polysaccharide synthesized from fructose 6-phosphate by several bacterial species. The aim of this study was to identify genes that are necessary for obtaining a normal level of alginate production in alginate-producing *Pseudomonas fluorescens*.

**Results:**

Polysaccharide biosynthesis is costly, since it utilizes nucleotide sugars and sequesters carbon. Consequently, transcription of the genes necessary for polysaccharide biosynthesis is usually tightly regulated. In this study we used an engineered *P. fluorescens* SBW25 derivative where all genes encoding the proteins needed for biosynthesis of alginate from fructose 6-phosphate and export of the polymer are expressed from inducible *Pm* promoters. In this way we would avoid identification of genes merely involved in regulating the expression of the alginate biosynthetic genes. The engineered strain was subjected to random transposon mutagenesis and a library of about 11500 mutants was screened for strains with altered alginate production. Identified inactivated genes were mainly found to encode proteins involved in metabolic pathways related to uptake and utilization of carbon, nitrogen and phosphor sources, biosynthesis of purine and tryptophan and peptidoglycan recycling.

**Conclusions:**

The majority of the identified mutants resulted in diminished alginate biosynthesis while cell yield in most cases were less affected. In some cases, however, a higher final cell yield were measured. The data indicate that when the supplies of fructose 6-phosphate or GTP are diminished, less alginate is produced. This should be taken into account when bacterial strains are designed for industrial polysaccharide production.

**Electronic supplementary material:**

The online version of this article (doi:10.1186/s12864-016-3467-7) contains supplementary material, which is available to authorized users.

## Background

Linear polysaccharides composed of mannuronic and guluronic acid residues that may be *O*-acetylated, are denoted alginate. These polymers are synthesized by brown and some red algae and by bacterial species belonging to the genera *Azotobacter* and *Pseudomonas*. Alginates manufactured from brown algae are currently used in diverse industrial and pharmaceutical applications. However, alginates produced by bacteria can more easily be tailored to obtain the compositions desired for the more high-value end of the alginate market [1], and this has motivated our studies on alginate-producing bacteria.

Production of a secreted polysaccharide imposes a drain on the cell’s carbon and energy sources, and thus the biosynthesis is usually tightly regulated under natural conditions. In batch cultures, alginate-producing *P. fluorescens* mutants display a reduced cell yield compared to the corresponding non-alginate producing strains [2]. Bacterial alginate production is controlled by the alternative sigma factor AlgU and is usually turned off in *Pseudomonas spp.* Induction of alginate biosynthesis results in a proteolytic cascade that finally cleaves the AlgU anti-sigma factor MucA, leading to transcription of the genes in the *alg* operon [3].

**Fig. 1:**
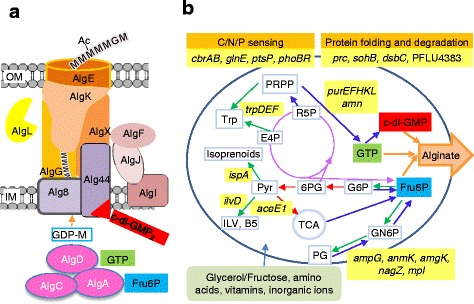
The relationship between alginate biosynthesis and the cellular metabolism in *P. fluorescens*. **a** The proteins and metabolites needed for alginate biosynthesis. **b** A simplified model of the cell’s metabolism highlighting the processes identified in the present study as being important for full alginate biosynthesis levels. The genes discussed in the paper are highlighted in *yellow*. The Entner-Doudoroff pathway and the oxidative part of the pentose phosphate pathway are indicated by *red arrows*, and the non-oxidative part of the pentose phosphate pathway with *purple arrows*. *Green arrows* indicate other pathways competing with accumulation of the three metabolites Fru6P, GTP and c-di-GMP, while blue arrows indicate pathways that would increase the synthesis of one of these three metabolites. Each arrow may represent several enzymatic steps. Abbreviations: OM: Outer membrane, IM: Inner membrane, M: mannuronic acid residue, G: guluronic acid residue, Ac: Acetyl, TCA: Tricarboxylic acid cycle, PP: the non-oxidative part of the pentose phosphate pathway, GN6P: Glucosamine 6-phosphate, PG: Peptidoglycan, G6P: Glucose 6-phosphate, 6PG: 6-phosphogluconate, Pyr: Pyruvate, ILV: Isoleucine Leucine Valine, B5: Pantothenate, Trp: Tryptophan, PRPP: Phosphoribosyl pyrophosphate, R5P: Ribose 5-phosphate, E4P: Erythrose 4-phosphate

In the first steps of bacterial alginate biosynthesis fructose 6-phosphate (Fru6P) is converted to GDP-mannuronic acid by the concerted action of AlgA, AlgC and AlgD. GDP-mannuronic acid is then polymerized to polymannuronic acid by Alg8 and the copolymerase Alg44. Together with AlgG, AlgX, AlgK and AlgE these form a protein complex that transports the alginate out of the cell as depicted in Fig. 1a [4]. AlgG also epimerizes some M-residues to G, while AlgI, AlgJ, AlgF and AlgX are needed to *O*-acetylate some of the M-residues. The alginate lyase AlgL removes alginate molecules that have been released to the periplasm [5]. Twelve of the thirteen genes directly involved in alginate biosynthesis are found in the *alg* operon, while the last, *algC*, is found elsewhere on the chromosome. This gene organization is found in all characterized alginate-producing bacteria. In addition to Fru6P and GTP, dimeric cyclic di-GMP (c-di-GMP) is needed for bacterial alginate biosynthesis [6, 7].

Recently we showed that the alginate synthesis rate is not proportional to the number of alginate biosynthetic complexes, indicating that there must be some kind of metabolic control as well [4]. In a recent transposon screen, some genes affecting AlgU-regulation were identified in *P. aeruginosa* [8]. However, the aim of the present study was to identify genes and pathways that influence alginate biosynthesis indirectly by perturbing the cell’s metabolism. An alginate-producing *P. fluorescens* strain in which the *alg* operon and *algC* is under control of the inducible *Pm* promoter was constructed and subjected to transposon mutagenesis. The *Pm* promoter and its activator XylS originally controls expression of the genes of the meta-cleavage pathway of aromatic hydrocarbons on the *Pseudomonas putida* plasmid pWW0 [9]. We have earlier shown that the *Pm* promoter and the weaker *Pm* promoter derivative *Pm-G5* are useful for obtaining different levels of controlled gene expression in *P. fluorescens* [5]. About 11500 insertion mutants were screened with respect to growth and alginate biosynthesis, and the inactivated genes in mutants displaying altered alginate yields were identified. The results supported our hypothesis that further levels of post-translational regulation exist, allowing the cell to prioritize basic cellular metabolism over alginate biosynthesis.

## Results and discussion

### Construction of a *P. fluorescens* strain in which the alginate biosynthesis genes are controlled by the inducible *Pm* promoter

**Fig. 2:**
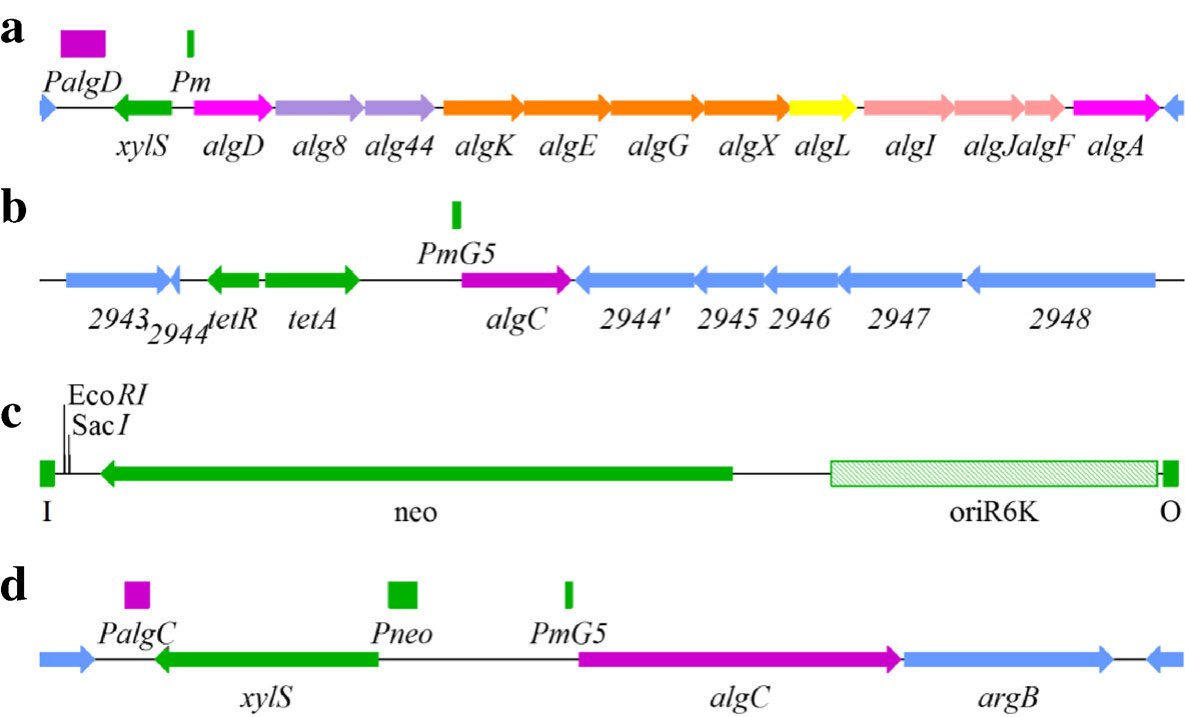
Genotypes for selected genetic constructs used in this study. **a** Strain MS1 in which the *Pm* promoter and the gene encoding XylS is inserted between the promoter and start codon of *algD*. **b** Strain MS2 in which a transposon expressing *algC* from *PmG5* is inserted into PFLU2944 in an *algC* ^−^ derivative of MS1. **c** Map of the transposon TnMS11 used for mutagenesis in this study. **d** Strain HE230 in which the gene encoding XylS and the *PmG5* promoter is inserted between the promoter and start codon of *algC* in SBW2*5mucA*. Inactivation of *mucA* confers a high level expression from wild type *PalgD*. Relevant promoters, and the two restriction sites used for sequencing are displayed above each map-line. The *alg*-genes are coloured to match Fig. 1, other *P. fluorescens* genes flanking the genes of interest are coloured *blue*, and heterologous genes and elements are coloured *green*. I and O denote the minitransposon ends

In order to avoid re-identification of the genes already known to directly regulate expression of the structural alginate biosynthetic genes, a derivative of *P. fluorescens* SBW25 designated strain MS1 was constructed (Fig. 2a). In this strain the naturally regulated *algD* promoter (which controls expression of the *alg* operon) was substituted with the wild-type *Pm* promoter. *xylS*, encoding the activator protein needed for expression from the *Pm* promoter, was inserted upstream of *Pm*. Then *algC* was inactivated by an in-frame deletion followed by a chromosomal insertion of a transposon containing a new *algC* copy expressed from a mutant version of *Pm* (*PmG5*) [5, 10]. This strain, designated MS2, produces only a small amount of alginate in the absence of *Pm* induction due to the low uninduced activity of *PmG5*.

Alginate production has been reported to affect cell yield in *P. fluorescens* [2], and it was also possible that m-toluic acid would have an effect on growth. This was tested by cultivating the non-alginate producing wild type strain SBW25 and strain MS2 in Biolector® for three days in 0.5 x PIA supplemented with glycerol as carbon source. Growth rate and cell yield was significantly lower for the induced strain MS2 relative to the non-alginate producing strain, while no effect was seen by cultivating SBW25 in the presence or absence of 0.5 mM m-toluic acid (Additional file 1: Figure S1).

The transposon carrying *algC* was found to disrupt PFLU2944, which is the last gene in an operon encoding a putative ABC transporter (Fig. 2b). In the presence of the *Pm*/*PmG5* inducer (*m*-toluate), the alginate production of strain MS2 was similar to that of strain MS1 (results not shown).

### Construction of a transposon insertion library and screening with respect to alginate synthesis

The transposon-containing suicide vector pMS11 (Fig. 2c) was used for mutagenesis of strain MS2. Nearly 11500 insertion mutants were picked robotically from the original agar medium plates and cultivated in 96-deep-well microtiter plates containing 0.5x liquid PIA with glycerol and *m*-toluate. After three days, cell densities and alginate production were measured. The initial screen was followed by a rescreen of primary candidates and 184 mutants were found to produce less than 50% (163 mutants) or more than 110% alginate (21 mutants) when compared to the parent strain. The transposon insertion sites in all these mutants were determined by DNA sequencing, leading to identification of 134 different genes belonging to most of the main cellular functions (results not shown). Of these genes only ten were known alginate biosynthesis structural genes, while one was *xylS*, the positive regulator of *Pm* expression. These results show that about 92% of the identified genes are not directly associated with alginate synthesis. The screen did not cover all relevant genes in the genome, since insertions in *algG, algF* and *algI* (members of the *alg* operon) were not found.

### Evaluation of the mutants to select candidates for further studies

Sequenced mutants with altered alginate phenotypes were cultivated in triplicates in 96-deep-well microtiter plates in three different media; 0.5xPIA with glycerol and 0.5xDEF4 with fructose or glycerol as carbon sources (7 g/L), and 0.5 mM *m*-toluate to induce alginate production. In the DEF4 media ammonium is the only nitrogen source, while PIA contains peptone that may be used as both nitrogen and carbon source. Furthermore, DEF4 contains more phosphate than PIA. The alginate yield from the control strain (MS2) was significantly higher in the DEF4 media, about 3 g/L compared to about 1 g/L in PIA, which resulted in better accuracy of the data in DEF4 for low alginate producers.

Results for mutants displaying significantly altered alginate production levels in at least one of the three media, are shown in Table 1. Significant changes were defined as less than 50% or more than 110% of the alginate production of the parent strain, and 36% of the retested gene-inactivation mutants did not meet this criterion. No mutant produced more alginate than the control strain in all three media. Mutants with insertions in alginate biosynthetic genes and *xylS* did, as expected, not produce alginate and are not included in Table 1. When several mutants had the same gene inactivated and displayed similar phenotypes, results from only one of them are shown in Table 1. For mutants where genes involved in glycerol utilization, amino acid biosynthesis or phosphate uptake had been inactivated, one would expect that the observed effects on biomass and alginate yield should be media dependent. As shown in Table 1 this was the case for most genes belonging to these categories.

**Table 1:** Identified mutants and their growth yield and alginate production in the three media^a^

Number of independent transposon mutants	Gene ID	Gene	Function	Growth (G) and Alginate production (A) in different media^a^											
				PIA Gly				0.5xDEF4 Gly				0.5xDEF4 Fru			
				G		A		G		A		G		A	
				%	SD	%	SD	%	SD	%	SD	%	SD	%	SD
	SBW25 WT			**132**	6	***0***	*25*	86	0	***0***	0	**350**	6	***1***	6
	Control			100	6	100	13	100	2	100	13	100	5	100	3
1	PFLU0460	*aceE1*	Energy production and conversion	66	2	63	2	57	4	*19*	4	**293**	20	***0***	4
1	PFLU3193	*aceE2*		108	4	*48*	2	98	6	89	9	**130**	3	106	19
1	PFLU5345	*cioB*		**151**	18	69	3	102	1	65	5	**129**	7	**119**	13
2	PFLU3801	*ftsK*	Cell cycle control, cell division, chromosome partitioning	*38*	0	*17*	5	72	14	***4***	3	**425**	42	***3***	3
	PFLU3801	*ftsK*		53	7	*39*	8	102	2	86	8	*16*	1	***7***	4
1	PFLU5304			107	6	*47*	7	100	6	96	8	**130**	18	101	12
1	PFLU1384		Amino acid transport and metabolism	108	3	*18*	3	71	1	81	33	**148**	45	95	6
1	PFLU2019			**126**	27	***0***	3	116	14	***0***	1	**338**	14	***0***	0
1	PFLU2124			**127**	4	*40*	7	102	2	88	7	**294**	30	55	27
1	PFLU3475			106	2	*45*	11	98	7	88	9	**132**	5	99	32
1	PFLU3887			104	4	*12*	11	93	6	72	16	**132**	28	53	5
1	PFLU5797	*ilvD*		69	22	64	2	60	10	*17*	1	**338**	31	***2***	3
1	PFLU4188	*trpF*		100	2	*34*	3	105	5	***0***	0	**343**	3	***0***	5
1	PFLU5559	*trpD*		62	5	58	11	106	9	***9***	12	**344**	23	***0***	1
2	PFLU5561	*trpE*		102	2	55	4	**140**	1	***0***	3	***4***	14	***1***	2
1	PFLU0612	*purH*	Nucleotide transport and metabolism	*44*	5	***4***	2	81	3	***0***	0	**317**	13	***0***	0
1	PFLU4183	*purF*		55	2	*48*	21	81	2	***9***	6	**344**	11	***4***	2
4	PFLU5034	*purL*		50	1	*43*	10	70	1	***1***	3	**348**	7	***0***	2
2	PFLU6054	*purK*		84	4	***2***	5	113	8	***0***	4	**343**	3	***1***	4
1	PFLU6055	*purE*		88	7	*45*	25	87	5	*37*	5	**368**	9	***0***	2
2	PFLU5396	*amn*		79	2	*29*	17	106	2	66	9	**135**	16	62	18
2	PFLU1142	*glpK*	Carbohydrate transport and metabolism	52	2	66	39	*19*	7	***2***	4	**214**	9	52	40
3	PFLU1143	*glpF*		59	2	***4***	2	*18*	3	***0***	2	**128**	10	80	4
1	PFLU3030	*paaF*		109	5	*33*	7	94	4	84	18	122	7	**118**	16
2	PFLU3365	*treZ*		107	2	62	12	97	1	98	3	**132**	17	**132**	4
3	PFLU4630	*acnA*		**144**	8	73	14	92	7	68	23	125	3	**113**	20
1	PFLU4949	*pykA*		107	4	*41*	14	51	3	**132**	5	**160**	3	85	12
1	PFLU0416	*hemE*	Coenzyme transport and metabolism	**147**	5	68	8	98	2	66	14	117	11	**136**	12
1	PFLU5820	*nudH*	Translation, ribosomal structure and biogenesis	77	3	*23*	12	78	0	**127**	5	65	23	*31*	3
1		*23S rRNA*		**152**	10	52	16	98	1	94	14	**130**	15	**142**	24
1	PFLU3173		Transcription	92	8	*10*	3	93	2	89	4	**276**	0	67	7
1	PFLU3307			**165**	18	74	11	103	2	88	28	**137**	10	**110**	8
1	PFLU4259			125	2	62	1	75	5	84	11	**153**	20	**111**	5
1	PFLU4774			102	10	***7***	3	91	12	75	7	110	3	79	26
1	PFLU5984	*dut*	Replication, recombination and repair	114	6	*45*	6	*47*	3	62	7	**149**	5	86	1
1	PFLU0013	*htrB*	Cell wall/ membrane/ envelope biogenesis	117	7	*40*	5	78	5	62	3	123	24	**110**	18
1	PFLU1562	*nagZ*		67	1	***5***	2	89	2	60	20	**186**	9	80	7
1	PFLU4993	*ampG*		58	2	***4***	2	87	1	53	10	**198**	6	83	17
1	PFLU5439	*mpl*		**129**	6	*36*	5	103	1	80	8	106	4	*46*	6
2	PFLU5545			62	3	***8***	2	99	9	90	11	**204**	1	65	6
2	PFLU5546	*anmK*		54	4	***3***	2	86	4	68	11	**192**	1	59	26
3	PFLU5573	*amgK*		95	30	*45*	10	*23*	2	*15*	4	**192**	18	*46*	38
1	PFLU5461	*ispA*		88	5	***2***	6	85	4	*46*	8	**282**	31	*15*	7
1	PFLU4418	*fleN*	Cell motility	88	1	*43*	4	80	4	104	29	80	1	*40*	12
1	PFLU4439	*fliF*		**140**	8	*12*	2	97	3	***0***	0	**342**	3	***0***	0
1	PFLU4448	*fliC*		111	8	*38*	2	87	5	102	7	**171**	3	**132**	6
1	PFLU0870	*tldD*	Posttransla-tional modification, protein turnover, chaperones	111	3	*26*	5	103	1	88	6	**137**	6	**111**	16
4	PFLU2032	*prc*		100	2	*37*	6	81	3	59	6	124	7	*26*	6
2	PFLU2614	*sohB*		98	3	*11*	3	96	1	73	8	108	7	109	12
1	PFLU3805	*clpA*		**147**	7	60	7	104	1	**127**	20	**256**	8	**124**	21
3	PFLU4383			118	6	*21*	8	87	22	51	5	**298**	8	*38*	13
1	PFLU5007	*dsbC*		102	7	*13*	6	97	2	79	5	120	10	84	2
2	PFLU5911	*ppx*	Inorganic ion transport and metabolism	50	2	82	38	73	3	67	28	90	13	76	2
	PFLU5911	*ppx*		54	1	***0***	0	73	4	101	7	107	5	98	15
1	PFLU0511	*rsgA*	General function prediction only	108	5	86	11	96	4	101	32	104	12	**124**	26
1	PFLU2104			**149**	9	66	18	94	1	65	13	117	7	**136**	15
1	PFLU2996			**134**	10	66	7	104	3	74	12	122	9	**132**	5
1	PFLU3202			108	3	76	10	81	3	*47*	6	**403**	7	*22*	3
1	PFLU3391			51	25	58	8	68	1	95	7	118	0	**129**	13
1	PFLU3411			105	6	50	9	101	2	70	29	**135**	2	**137**	19
1	PFLU3456			104	4	*47*	5	103	0	107	2	**151**	13	**117**	6
1	PFLU1883		Function unknown	69	0	***0***	0	74	14	66	14	**712**	268	***0***	0
1	PFLU1995			**149**	7	85	9	104	1	97	7	**158**	18	**113**	17
1	PFLU4517			**129**	3	82	6	96	3	*28*	5	**377**	18	***2***	6
1	PFLU5579	*apaG*		*39*	2	*47*	21	104	5	***4***	3	**175**	32	*18*	7
1	PFLU2489			93	10	**120**	51	69	3	75	8	**324**	40	*23*	9
1	PFLU5377			107	8	***8***	3	97	4	76	5	**145**	1	92	20
1	Upstream PFLU2629			107	6	***9***	3	106	4	84	13	**300**	16	106	7
1	Upstream PFLU3162			122	2	77	20	76	1	88	12	**280**	36	96	15
1	Upstream PFLU3931			97	2	*35*	3	75	2	***3***	3	**327**	11	*12*	5
1	PFLU2519		Pseudogene	114	4	**148**	26	102	3	98	6	**337**	9	89	13
1	PFLU0259	*ompR*	Signal transduction mechanisms	89	5	***7***	1	101	4	70	12	**127**	17	101	12
2	PFLU0461	*glnE*		85	14	***10***	4	120	4	104	13	**317**	11	67	1
1	PFLU4125A			119	4	**164**	14	112	0	86	4	**158**	5	72	2
4	PFLU5236	*cbrA*		**140**	1	*34*	7	105	4	55	7	**319**	5	66	21
2	PFLU5237	*cbrB*		118	1	*15*	3	109	3	*23*	3	**483**	24	*31*	2
2	PFLU5819	*ptsP*		80	12	***8***	3	90	6	79	15	*49*	1	***5***	5
1	PFLU6039	*phoB*		78	1	*44*	20	102	2	90	16	110	7	84	21
1	PFLU6040	*phoR*		*25*	0	***0***	0	97	12	87	0	99	22	100	23
1	PFLU2808			100	2	*11*	3	110	4	75	5	**255**	52	*19*	11
1	PFLU3002		Intracellular trafficking, secretion, and vesicular transport	**153**	6	*48*	15	93	5	83	6	**141**	9	94	5
1	PFLU3951			106	6	***8***	1	105	2	83	1	110	5	101	20
1	PFLU5567			98	9	61	5	***0***	0	***0***	0	59	1	***2***	4

a: The strains were cultivated in microtiter plates for three days before cell and alginate yield were measured. The mutants shown are those that displayed significantly different alginate production levels in at least one of the three tested media. Data are not shown for strains with transposon insertions in the genes encoded by the alginate operon or in *algC*. The Table shows how many independent transposon insertions mutants that were identified for each gene, the gene identifier, the gene name, and which functional group the corresponding protein is assigned to. Growth above 125% and alginate production above 110% are marked using bold types, growth and alginate production between 10 and 50% are marked using italics, and growth and alginate production below 10% are written in bold italics. Three biological replicates were cultivated for each strain, and the results are given as percent (%) of the values obtained from the control strain MS2. Standard deviations for the three replicates are shown in the columns to the right (SD)

It is probable that in many cases the phenotype observed in a transposon insertion mutant is caused directly by inactivation of the identified gene. However, polar effects (particularly in operons) and unrelated, spontaneous mutations can certainly not be excluded. For those genes where several independent transposon insertion mutants were identified, it is more likely that the observed phenotype is caused by the observed transposon insertion. The same argument may be used when several genes encoding proteins in the same metabolic pathway have been identified. In addition, 18 of the identified genes were chosen to be complemented either by expressing the wild type gene on a transposon or by adding the lacking metabolite. The transposons were constructed and transferred to the mutant strains, and both the mutant strains and the complemented strains were cultivated in two new growth experiments (Tables 2 and 3). Two of the 18 mutants could not be complemented and are not discussed further. These results show that the phenotypes of 16 out of 18 (89%) tested mutants can be explained by the transposon insertions only.

**Table 2:** Growth and alginate production of mutants using medium supplements or complementing transposons^a^

Inactivated gene	Supplement/comple-menting gene (s)^b^	PIA		0.5xDEF4 Glycerol		0.5xDEF4 Fructose	
		Growth^c^	Alginate	Growth^c^	Alginate	Growth	Alginate
wt		100	100	100	100	100	100
*trpF*		65	***0***	**145**	***0***	**291**	*38*
	tryptophane	70	**139**	78	60	**130**	*45*
	*trpF*	88	**121**	88	94	**153**	106
*trpD*		56	***0***	***1***	***0***	***3***	***0***
	tryptophane	68	**261**	68	80	71	75
	*trpD*	85	**142**	114	*23*	**242**	*38*
	*trpDC*	89	85	81	91	**152**	**120**
*purH*		*29*	***0***	***1***	***0***	*21*	***0***
	Adenine, thiamine	51	**188**	*14*	*33*	*39*	*21*
	*purH*	90	100	91	88	**163**	97
*purE*		*33*	***0***	***0***	***0***	***1***	***0***
	Adenine, thiamine	56	91	*17*	*50*	*43*	*29*
	*purE*	57	***0***	***1***	***0***	***1***	***0***
*purL*		*19*	***0***	***0***	***0***	***1***	***0***
	Adenine, thiamine	52	**124**	*12*	*48*	*38*	*30*
*ilvD*		77	*18*	***2***	***2***	***4***	***0***
	*ilvD*	91	82	91	109	**222**	97
*aceEI*		*33*	58	***6***	*20*	*13*	***7***
	*aceEI*	96	*42*	85	96	**244**	**123**
PFLU3030		88	***0***	80	89	104	103
	PFLU3030	97	**142**	89	**123**	101	**111**
*dsbC*		108	52	111	89	**149**	**115**
	*dsbC*	105	**127**	105	**117**	94	92
*sohB*		100	***9***	100	*50*	**479**	***−2***
	*sohB*	91	109	104	106	109	100
*nagZ*		53	***0***	**139**	93	115	75
	*nagZ*	87	**145**	84	**120**	**289**	79
*anmK*		*32*	***0***	124	98	**197**	99
	*anmK*	84	**118**	86	**135**	111	**128**
*ispA*		82	***0***	100	97	121	**115**
	*ispA*	92	**212**	88	**117**	**405**	77
*cbrB*		83	***0***	110	63	**374**	*46*
	*cbrB*	91	**118**	91	95	**213**	107
PFLU3887		91	67	98	103	105	**110**
	PFLU3887	90	*33*	109	103	112	103
PFLU5567		87	64	***2***	***6***	**141**	*30*
	PFLU5567	93	103	***1***	*16*	***6***	*10*

a: The strains were grown in deep-well plates containing the indicated media for four days before cell and alginate yield were measured. b: empty field denotes no supplement or complementing vector. c: Values are given as percentage of the control strain (SBW25 MS1 Δ*algC*:: TnKB61). Actual values for the control strain were (growth [OD_660_]/alginate [g/L]): PIA: 0.492/0.33, DEF4 glycerol: 0.850/1.72, DEF4 fructose: 0.308/3.08. Growth above 125% and alginate production above 110% are marked using bold types, growth and alginate production between 10 and 50% are marked using italics, and growth and alginate production below 10% are written in bold italics

**Table 3:** Effect of PhoBR disruptions on *P. fluorescens* growth and alginate biosynthesis

Strain	Growth (OD600)	Alginate (g/l)
SBW25*mucA*HE230	2.5+/−0.24	4.3+/−0.89
SBW25*mucA*HE230 Δ*phoR*	2.7+/−0.33	3.6+/−0.36
SBW25*mucA*HE230 Δ*phoB*	1.4+/−0.12	0.0+/−0.0
SBW25*mucA*HE230 Δ*phoR*:: TnTK5	2.4+/−0.09	4.0+/−0.24
SBW25*mucA*HE230 Δ*phoR*:: TnTK7	2.1+/−0.10	4.3+/−0.66
SBW25*mucA*HE230 Δ*phoB*:: TnTK6	1.5+/−0.20	1.2+/−0.56
SBW25*mucA*HE230 Δ*phoB*:: TnTK7	2.0+/−0.13	5.5+/−0.12

a: The cells were grown for 72 h in shaking flasks using DEF3 medium with 20 g/l glycerol, 1 μM phosphate and 0.5 mM m-toluate. Average values from three independent experiments are shown

Alginate biosynthesis requires a functional biosynthetic complex, Fru6P and a dimeric form of c-di-GMP (Fig. 1a). Interestingly, the majority of those mutants that reproducibly produced less alginate were assigned to the groups involved in uptake and metabolism of carbohydrates, amino acids and nucleotides (Table 1). In addition four genes encoding proteins involved in protein modification were identified. Fig. 1b summarizes how the pathways identified in the current study might influence alginate yield, and these genes and pathways are discussed in more detail below.

### Alginate production is influenced by signal transduction systems involved in carbon, nitrogen and phosphor metabolism

Four different signal transduction systems, CbrAB, NtrBC, PTS^Ntr^, and PhoBR, were identified in the screen by using the criteria of either complementation or identification of several independent mutants in specific genes or pathways. The CbrAB two-component system has been described in several species of *Pseudomonas* as sensors and regulators of genes involved in utilization of different carbon and nitrogen sources, and has been proposed as sensors for the C/N balance in the cell [11, 12]. It has been shown that CbrB activates the expression of non-coding RNAs that relieve the catabolite repression otherwise exerted by Crc [13]. In *P. putida*, inactivation of *cbrB* also affected stress responses and biofilm development [14]. Our results show that the identified *cbrB* mutant produces less alginate (0-63%) than the otherwise isogenic control strain in all three media (Table 2). The mutant could be complemented by introducing a transposon-encoded copy of *cbrB* (Table 2). The effect of inactivating *cbrA* was, however, less pronounced, and might be caused by a polar effect on *cbrB* (Table 1). In *P. putida*, a *cbrB* mutant was shown to be unable to use some amino acids as carbon source, and to have an increased expression level of some of the genes encoding proteins involved in the Entner-Doudoroff pathway [14]. If the consequences of inactivating *cbrB* is similar in *P. fluorescens*, these two effects alone might explain the observed growth and alginate yields for the *cbrB* mutants, by reducing the net flow to Fru6P (Fig. 1b). However, given the known pleiotropic nature of a *cbrB* mutation, this probably is not the full explanation.

NtrBC is known to be an important response regulator system for bacterial nitrogen sensing, and has been found to interact with the CbrAB system [14]. GlnE is needed for the posttranscriptional activation of glutamine synthase, which is a part of the NtrC regulatory cascade [15]. It has been shown that inactivation of this gene lowered the pool of Fru6P in *Corynebacterium glutamicum* [16]. Consistent with this the alginate yield was significantly lower in PIA and in DEF4 with fructose for both *glnE* mutants (Table 1).

Glutamine and α-ketoglutarate are used by the NtrC-cascade to sense the carbon and nitrogen status of the cell, and these metabolites were recently found to affect the phosphorylation rate of the nitrogen-related phosphoenolpyruvate phosphotransferase system (PTS^Ntr^) in *E. coli* [17]. PTS^Ntr^ is also known to form a link between carbon and nitrogen metabolism in pseudomonads [18]. While fructose is probably imported and phosphorylated by a PTS in *P. fluorescens*, glycerol is taken up through a transport and kinase system and is fed into the central metabolism as triose phosphates [19]. PtsP (EI^Ntr^) is the first protein in the nitrogen-related phosphate relay, and the two *ptsP* mutants identified in the current study produced low amounts of alginate both in PIA (24 and 8%) and in DEF4 with fructose (14 and 5%). An earlier study has shown that a *ptsP* mutant of *P. putida* produces less polyhydroxyalkanoate than the wild type, and it was suggested that such a mutant would behave as if there was a carbon limitation [20]. A similar argument could be used to explain the lower yield of alginate in our *ptsP* mutant. Recently it was also shown that inactivation of *ptsP* in *P. aeruginosa* decreases the level of c-di-GMP [21].

The response regulator PhoB and the histidine kinase PhoR control the Pho-regulon, which covers a major pathway for bacterial adaptation to phosphate starvation. PhoB may also be activated by other kinases [22]. Since *phoB* and *phoR* form an operon, new in-frame deletion mutants for each of these genes were constructed in the alginate-producing strain SBW25*mucA*HE230 (Fig. 2d). This strain was chosen because our standard gene recombination vector could not be used in the tetracycline-resistant strain MS2. The wild-type genes were cloned both individually and as an operon on transposons, and these transposons were used to complement the deletion mutants. The new *phoR* mutant behaved similarly to the wild type strain, while the *phoB* deletion resulted in lower cell yield and no alginate production when cultivated in DEF3 with reduced phosphate concentration (1 μM) (Table 3). Both traits were restored by chromosomal insertion of a transposon encoding both *phoB* and *phoR*, while chromosomal insertion of a transposon encoding *phoB* only partially regained alginate production and normal growth. Lack of PhoB will lead to decreased phosphate uptake under phosphate-limiting conditions, and this may result in less trinucleotides [23]. Furthermore, in *Pseudomonas aeruginosa* the AlgQ (AlgR2), has been shown to regulate the production of GTP through its positive regulatory effect on transcription of *ndk*, and Ndk is required for alginate production [24]. AlgQ is an anti-sigma-70 factor and has been shown to positively regulate alginate production [25], possibly by increasing the amount of RNAP available for the alternative sigma-factor AlgU. Transcription of *algQ* is positively regulated by PhoB [24]. In our strain, transcription of the alginate biosynthetic genes depends on the *Pm* promoter, which in turn depends on the sigma factors RpoH and RpoS for transcription [26]. Thus, it is possible that AlgQ may have a positive effect on expression from *Pm*. If that is the case, this might also explain the lack of alginate production in the *phoB* mutant when grown in a low phosphate medium.

### Inactivation of certain genes involved in cell wall metabolism and vitamin biosynthesis leads to decreased alginate yield

In the present screen, insertions in five of the nine genes known to be involved in peptidoglycan recycling in *Pseudomonas* [27] were identified as having a negative impact on alginate biosynthesis (*mpl, ampG, anmK, amgK and nagZ)*. The absence of Mpl, which is involved in recycling of the peptide part of peptidoglycan, only slightly decreased the alginate production. However, absence of any of the other four identified enzymes, AmpG, AnmK, AmgK or NagZ, resulted in very low alginate production in the PIA medium and reduced alginate yield in the DEF4 media (Table 1). The sugar phosphates used for peptidoglycan synthesis either originates from peptidoglycan recycling or is synthesized from Fru6P (Fig. 1b). Since Fru6P is also a precursor for alginate, depletion of this phosphorylated sugar would be expected to cause decreased alginate yield [2]. The *nagZ* and *anmK genes* were cloned on transposons, and shown to complement the deficiency in alginate production in the corresponding insertion mutants (Table 2).

Three of the identified genes, *aceE1, ilvD* and *ispA* were linked to pyruvate metabolism (Fig. 1b). *aceE1* encodes a component of pyruvate dehydrogenase, which is an essential part of the central carbon metabolism. The viability of this mutant might be explained by the presence of other genes encoding AceE-like proteins in *P. fluorescens.* However, the *aceE1* mutant grew more slowly than strain MS2, and hardly produced any alginate. *ilvD* encodes a dihydroxy-acid dehydratase that participates in the biosynthesis of branched amino acids and in the biosynthesis of pantothenate (vitamin B5) and coenzyme A. The *ilvD* mutant displayed a similar phenotype as the *aceE1* strain in all three media (Table 2). The *ispA* mutant would be expected to have defects in the biosynthesis of isoprenoids, which would affect the biosynthesis of ubiquinone and the cell membrane. This mutant produced very low amounts of alginate when grown in PIA, while the phenotypes in the DEF4 media were more similar to the control strain (Table 2). All three mutants were complemented when the corresponding wild type genes were expressed from transposons (Table 2). Disruption of a pathway may often result in an increased flow to the immediate precursor for the missing enzyme, since the cell will perceive a lack of the end product. In the *ispA* and *ilvD* mutants this would lead to consumption of pyruvate, which then would have to be replenished by increasing the flow through the Entner-Doudoroff pathway (Fig. 1b). Pantothenate (needed for CoA) and ubiquinone are necessary for the anabolism and energy production of the cell, and the medium-dependent defects in growth and alginate yield displayed by the mutants might be caused by a lower content of these vitamins in peptone (PIA) compared to yeast extract (DEF4).

### Deficiencies in purine or tryptophan biosynthesis reduce alginate yield

Eleven of the mutants identified in the screen turned out to have insertions in genes needed for purine biosynthesis (*purHFLKE* and *amn*). GTP is required for alginate biosynthesis as a precursor for both GDP-mannuronic acid and the signal molecule c-di-GMP (Fig. 1b). Three of the identified purine biosynthesis mutants (*purE, purH* and *purL)* were retested in deep-well plate cultivations and grew poorly in all media (Table 2). The *purH* strain was complemented when wild-type *purH* was expressed from a transposon, while the *purE* mutant was not complemented by expressing *purE*. This might, however, result from a polar effect on the downstream *purK* gene. Addition of adenine and thiamine to the media increased both growth and alginate yield for all three mutants (Table 2), strongly suggesting that the observed phenotypes were caused by deficiencies in the purine synthesis pathway.

In eight of the sequenced mutants, the transposon had disrupted genes putatively involved in amino acid biosynthesis (Table 1). Three of these, *trpDEF,* were genes involved in tryptophan synthesis. The mutants with insertions in *trpD* and *trpF* were investigated further and both could be complemented by inserting an intact corresponding gene on a transposon (Table 2). Furthermore, addition of tryptophan to the growth medium restored normal growth and alginate yield in both mutants (Table 2).

Both tryptophan and purine synthesis are linked to Fru6P through the pentose phosphate pathway (Fig. 1b). Defects in these biosynthetic pathways might affect alginate synthesis negatively by increasing the need for phosphoribosyl pyrophosphate (PRPP), and thus increase the flow from Fru6P to this intermediate. Since GTP is necessary for alginate biosynthesis, the observed phenotypes might also be caused by an insufficient supply of purines. Our results are corroborated by other studies demonstrating that *de novo* synthesis of purine is necessary for biofilm formation in *P. fluorescens* [28], and that tryptophan is important for biofilm formation in *Salmonella enterica* [29].

### Disruption of several genes encoding proteins involved in protein folding and modification result in reduced alginate yield

Prc is a protease known to affect alginate biosynthesis in some *mucA* mutants of *P. aeruginosa*, and has been proposed to indirectly participate in alginate biosynthetic gene activation through MucA cleavage induced by cell wall stress [30, 31]. However, in our strain both *algC* and the *alg* operon are controlled by the *Pm* promoter, not by the endogenous AlgU-MucA-regulated promoters. Still, four independent *prc* mutants were identified as displaying a reduced alginate yield (Table 1). Our results therefore show that in *P. fluorescens* a *prc* mutation negatively affects alginate biosynthesis even in a *mucA* ^*+*^ strain. In addition the screen identified another peptidase belonging to the same family, SohB, which also negatively affected alginate yield when inactivated. This phenotype was complemented by a transposon expressing *sohB* (Table 2). It is unknown which proteins, apart from MucA, is the target of these two proteases in *P. fluorescens*.

Two genes encoding proteins involved in protein folding were identified in the screen as producing less alginate than the control (Table 1). PFLU4383 encodes a putative thiol:disulfide interchange protein and is located upstream of and partly overlapping *dsbG*, encoding another disulfide isomerase. Three independent inactivations of PFLU4383 were identified. PFLU5007 encodes the disulfide isomerase DsbC and its phenotype was complemented by a transposon-encoded copy of the gene (Table 2). A mutant of *P. aeruginosa* with transposon-inactivated *dsbC* was recently found to display a non-mucoid phenotype [32], indicating that DsbC is needed for normal levels of alginate production in both species. The results suggest that full alginate production in these media depend on correct folding of some proteins. It remains unknown which proteins need these isomerases for correct folding.

## Conclusion

In an earlier study, it was shown that inactivation of glucose-6-phosphate dehydrogenase increased alginate yield when glycerol was used as carbon source, and this indicated that the availability of Fru6P may be one limiting factor to sustain high level alginate production [2]. Furthermore, it has been shown that the number of alginate biosynthetic complexes are not influenced by the absence of precursors for alginate synthesis [4], indicating that these complexes are not destabilized in the absence of polymer synthesis. The aim of screening a transposon insertion library, was to discover genes and metabolic pathways that indirectly influence alginate production in *P. fluorescens*. The main conclusion of our data is that alginate biosynthesis depends on sufficient levels of Fru6P, GTP and c-di-GMP (Fig. 1b). Inactivation of genes in several systems sensing the carbon/nitrogen ratio resulted in mutants that produce less alginate than the parent strain, and this further indicates that alginate production might be down-regulated as a response to a perceived carbon limitation. A majority of the analysed mutants displayed a significantly decreased alginate yield, while the cell yield was less affected, and in some cases even increased. This suggests that when *P. fluorescens* is facing certain nutrient limitations, less alginate is produced.

## Methods

### Growth of bacteria

*E. coli* and *P. fluorescens* (Table 4) were routinely cultivated in L broth or on L agar at 37 °C or 30 °C, respectively [33]. *P. fluorescens* was also grown in PIA medium [33], DEF4 medium [34] and DEF3 medium with low phosphate: KH_2_PO_4_ 0,14 mg/L, KCl 0.36 g/L, NH_4_Cl 2.21 g/L, citric acid · H_2_O 0.9 g/L, ferric citrate 0.02 g/L, H_3_BO_3_ 0.001 g/L, MnCl_2_ · 4H_2_O 0.005 g/L, EDTA · 2H_2_O 0.0039 g/L, CuCl_2_ · 2H_2_O 0.0005 g/L, Na_2_Mo_4_O_4_ · 2H_2_O 0.0008 g/L, CoCl_2_ · 6H_2_O 0.0008 g/L, Zn (CH_3_COO)_2_ · 2H_2_O 0.0027 g/L, NaCl 1.56 g/L, MgSO_4_ · 7H_2_O 0.57 g/L, MOPS 10 g/L. For precultures, 0.39 g/L yeast extract was added to the DEF4 medium. The pH of DEF3 and DEF4 was adjusted to 7.0. Carbon sources – fructose or glycerol – were added to 20 g/L. Antibiotics used: ampicillin (Ap, 200 mg/L), tetracycline (Tc, 15 mg/L), apramycin (Am, 25 mg/L), kanamycin (Km, 50 mg/L). For growth in microtiter plates and micro bioreactors (BioLector®), half the concentrations of the media containing 7 g/L carbon source was used, and the cultures were incubated at 25 °C as detailed previously [34]. For some experiments adenine (0.8 mM), thiamine (0.05 mM), or tryptophan (2.5 mM) were added as medium supplements. For growth studies in Biolector® microreactors the cultivations were performed in M2P-labs FlowerPlate® BOH with 1 ml medium per reactor. The cultivations were started (3 vol-% inoculum) from L broth precultures cultivated at 30 °C for 18 h. The BOH plates were incubated at 25 °C, 1300 rpm with 3 mm orbital movement at 80% humidity. pH, dissolved oxygen and biomass were measured automatically every hour by the Biolector system. The biomass measured by the Biolectors Photomultiplier was calibrated by offline optical density measurements using a standard spectrophotometer.

**Table 4:** Bacterial strains^a^ and plasmids used in this study

Strains	Description	Reference
*E. coli* S17-1 (λpir)	*λpir* (for replication of oriR6K-plasmids) *recA, thi pro hsdR-M* ^*+*^ RP4 2-Tc::Mu-Km::*Tn7*TpRSMR	[36]
*P. fluorescens* SBW25	Non-mucoid *P. fluorescens* wild type	[37]
SBW25MS1	Derivative of SBW25 where the *Pm* promoter is inserted directly upstream of *algD* using pMS9.	This study
SBW25MS1 Δ*algC*:: TnKB61	Derivative of SBW25 MS1 where *algC* has been deleted utilizing pKB22, and a copy of *algC* controlled by the *PmG5* promoter has been introduced on a transposon inserted into gene *PFLU2944*.	This study
SBW25*mucA*HE230	Alginate-producing derivative of SBW25 encoding a defect MucA and where the expression of *algC* is controlled by the *PmG5* promoter	H. Ertesvåg, unpublished
SBW25*mucA*HE230Δ*phoB*	Derivative of SBW25*mucA*HE230 where an in-frame deletion in *phoB* was introduced utilizing pTK10.	This study
SBW25*mucA*HE230Δ*phoR*	Derivative of SBW25*mucA*HE230 where an in-frame deletion in *phoR* was introduced utilizing pTK9.	This study
**Plasmids**		
pKD20	pUT based transposon vector containing *PmG5*. Ap^r^, Km^r^.	[5]
pLitmus28Tc	High copy number cloning vector. Tc^r^, Ap^r^	[5]
pMG48	RK2-based gene replacement vector. *lacZ* ^+^ *,* Tc^r^, Ap^r^	[33]
pMC1	RK2-based gene replacement vector for replacing the DNA sequence upstream of *algD* with the *Pm*-promoter. *lacZ* ^+^ *,* Tc^r^, Ap^r^	[10]
pKB22	Gene replacement vector for creating an *algC*-deletion. *lacZ* ^+^ *,* Tc^r^, Ap^r^	[5]
pKB60	Transposon vector. Contains the transposon TnKB60 with *algC* under the control of *PmG5*. Ap^r^, Km^r^	[5]
pYQ1	pUT based transposon vector containing *PmG5*. Am^r^, Km^r^.	[2]
pEM1	Derivative of pLitmus28Tc containing part of the transposon from pKD20. Ap^r^	[2]
pKB61	Derivative of pKB60 where a 1.7 kb AvrII-NcoI DNA fragment encoding Km^r^ and most of XylS was exchanged with a 2.5 kb AvrII-NotI DNA fragment containing *tetAR*. Tc^r^, Ap^r^	This study
pMS9	Derivative of pMC1 where a 0.7 kb SbfI-NotI DNA fragment containing a gene upstream of *algD* was exchanged with a PCR product containing the 0.8 kb sequence directly upstream of *algD. lacZ* ^+^ *,* Tc^r^, Ap^r^	This study
pMS2	Derivative of pLitmus28Tc where the *tetAR* genes were exchanged with a 3.4 kb BamHI-fragment from pKD20 containing the minitransposon and *oriR6K*. Km^r^, Ap^r^	This study
pMS10	Derivative of pMS2 where a 0.5 kb BsiWI-EcoRI-fragment containing *oriR6K* was deleted and the 1.9 kb NotI-PstI fragment encoding XylS was exchanged with a 0.4 kb PCR product encoding *oriR6K.* Km^r^, Ap^r^	This study
pMS11	Derivative of pKD20 where a 3.7 kb BssHII-SfiI-fragment was exchanged with a 1.5 kb BssHII-SfiI-fragment containing *oriR6K* from pMS10. Km^r^, Ap^r^	This study
pTK1	Derivative of pEM1 in which a 2.2 kb PCR-amplified NdeI-NotI DNA fragment encoding *phoBR* from *P. fluorescens* was inserted*.* Ap^r^.	This study
pTK3	Derivative of pTK1 in which an inserted 2.2 kb PCR-amplified NcoI-PspOMI DNA fragment from *P. fluorescens* including the first 46 nt of *phoB* replaced most of the *phoB* gene. Ap^r^.	This study
pTK4	Derivative of pTK1 from which a 0.9 kb BstEII-BsaBI DNA fragment encoding most of phoR was deleted. Ap^r^.	This study
pTK5	Derivative of pKD20 in which a 1.5 kb NdeI-NotI PCR fragment from *P. fluorescens* containing *phoR* was inserted. Km^r^.	This study
pTK6	Derivative of pKD20 in which a 1.1 kb NcoI-NotI DNA fragment from pTK4 containing *phoB* was inserted. Km^r^.	This study
pTK7	Derivative of pKD20 in which a 2.2 kb NdeI-NotI PCR fragment from *P. fluorescens* containing *phoBR* was inserted. Km^r^.	This study
pTK8	Derivative of pTK1 from which a 0.3 kb BstEII DNA fragment was deleted, creating an in-frame deletion in *phoR*. Ap^r^.	This study
pTK9	Derivative of pMG48 in which a 3.0 kb NcoI-NotI DNA fragment from pTK8 was inserted, containing a deletion in *phoR.* Ap^r^, Tc^r^.	This study
pTK10	Derivative of pMG48 in which a 3.1 kb NcoI-NotI DNA fragment from pTK3 was inserted, containing a deletion in *phoB.* Ap^r^, Tc^r^.	This study
pYQ1 trpF	Derivative of pYQ1 in which a 0.7 kb NdeI-NotI PCR fragment encoding TrpF was inserted. Am^r^.	This study
pYQ1 trpD	Derivative of pYQ1 in which a 1.1 kb NdeI-NotI PCR fragment encoding TrpD was inserted. Am^r^.	This study
pYQ1 trpDC	Derivative of pYQ1 in which a 1.9 kb NdeI-NotI PCR fragment encoding TrpDC was inserted. Am^r^.	This study
pYQ1 purH	Derivative of pYQ1 in which a 1.6 kb NdeI-NotI PCR fragment encoding PurH was inserted. Am^r^.	This study
pYQ1 purE	Derivative of pYQ1 in which a 0.5 kb NdeI-NotI PCR fragment encoding PurE was inserted. Am^r^.	This study
pYQ1 ilvD	Derivative of pYQ1 in which a 2.1 kb NdeI-NotI PCR fragment encoding IlvD was inserted. Am^r^.	This study
pYQ1 aceEI	Derivative of pYQ1 in which a 2.7 kb NdeI-NotI PCR fragment encoding AceE1 was inserted. Am^r^.	This study
pYQ1 PFLU3030	Derivative of pYQ1 in which a 1.0 kb NdeI-NotI PCR fragment encoding PFLU3030 was inserted. Am^r^.	This study
pYQ1 PFLU3887	Derivative of pYQ1 in which a 1.0 kb NdeI-NotI PCR fragment encoding PFLU3887 was inserted. Am^r^.	This study
pYQ1 PFLU5567	Derivative of pYQ1 in which a 1.2 kb NdeI-NotI PCR fragment encoding PFLU5567 was inserted. Am^r^.	This study
pYQ1 dsbC	Derivative of pYQ1 in which a 0.9 kb NdeI-NotI PCR fragment encoding DsbC was inserted. Am^r^.	This study
pYQ1 sohB	Derivative of pYQ1 in which a 1.1 kb NdeI-NotI PCR fragment encoding SohB was inserted. Am^r^.	This study
pYQ1 nagZ	Derivative of pYQ1 in which a 1.1 kb NdeI-NotI PCR fragment encoding NagZ was inserted. Am^r^.	This study
pYQ1 anmK	Derivative of pYQ1 in which a 1.5 kb NdeI-NotI PCR fragment encoding AnmK was inserted. Am^r^.	This study
pYQ1 ispA	Derivative of pYQ1 in which a 0.9 kb NdeI-NotI PCR fragment encoding IspA was inserted. Am^r^.	This study
pYQ1 cbrB	Derivative of pYQ1 in which a 1.4 kb NdeI-NotI PCR fragment encoding CbrB was inserted. Am^r^.	This study

a: Mutant strains complemented with transposons are not included in the Table

### Analyses of alginate and growth

The cultures were incubated for three to four days before the cell density and alginate yield were assayed. Enzymatic measurements of alginate production were performed as described earlier [2, 35]. Briefly, the cell free medium were treated with a mixture of an M-specific and a G-specific alginate lyase, and OD_230_ before and after the reaction were measured using a Beckman Coulter robotic liquid handling work station with a Paradigm microplate reader.

### Construction of the transposon vector and the transposon insertion library

Cloning, transformation, conjugation and gene deletions were performed as described earlier [33]. The plasmids and transposons used and constructed in this study are described in Table 4, while the primer sequences are found in Additional file 2: Table S1. PCR was performed using the Expand High Fidelity kit (Roche). PCR-amplified genes were confirmed by sequencing. Transposon insertions were to be identified by sequencing, so a transposon vector that would allow easy cloning of the transposon insertion site in *E. coli* was constructed and designated pMS11 (Table 4, Fig. 2c). The vector contains a derivative of the Tn5 minitransposon that comprises oriR6K and a gene encoding kanamycin resistance within the transposon boundaries. The transposon contains single sites for the restriction enzymes SacI and EcoRI close to the ends of the transposon. pMS11 was propagated in *E. coli* S17-1 λpir that encodes the Pir protein necessary for R6K-replication. pMS11 was transferred to *P. fluorescens* by conjugation, and conjugants were selected on PIA containing kanamycin. Colonies were picked using a Genetix QPixII colony picking robot and transferred to 384 well plates with 0.5 x PIA and Km, and incubated at 25 °C overnight before glycerol was added to 15% v/v and the plates were stored at −80 °C.

### Identification of transposon insertion sites

Genomic DNA was isolated from mutants of interest. For some mutants the transposon insertion site was identified by direct sequencing using this DNA as the template and the primer MS11 Ori (Additional Table S1). For sequencing on genomic DNA, 5 μg DNA, 50 pmol sequencing primer, 8 μl 2.5x BigDye Terminator Ready Reaction Mix v1.1 (Applied Biosystems) and water to 20 μl was mixed. The reaction was subjected to sixty cycles of 30 s denaturation at 95 °C, 30 s annealing at 52 °C, and four minutes elongation at 60 °C. Alternatively, the DNA flanking the transposon insertion site was cloned by restricting genomic DNA isolated from a transposon mutant with SacI or EcoRI. The fragments were circularized by ligation, and the ligation mixture was transformed into *E. coli* S17-1 λpir and selected for resistance to kanamycin. Sequencing the resulting plasmids provided better quality sequences than by sequencing directly on genomic DNA. The transposon insertion points were identified by comparing the obtained sequences to the genome sequence (GenBank Accession number AM181176).

## Additional file

Additional file 1: Figure S1. Growth profiles of *Pseudomonas fluorescence* SBW25 and MS2 cultivated in 0.5 x PIA. (PPTX 75 kb)
Additional file 2: Table S1. Primers used in the study. (XLS 30 kb)

## References

[1] Andersen T, Strand BL, Formo K, Alsberg E, Christensen BE, Rauter AP, Lindhorst TK (2012). Alginates as biomaterials in tissue engineering. Carbohydr Chem.

[2] Maleki S, Mærk M, Valla S, Ertesvåg H (2015). Mutational analyses of glucose dehydrogenase and glucose-6-phosphate dehydrogenase genes in *Pseudomonas fluorescens* reveal their effects on growth and alginate production. Appl Environ Microbiol.

[3] Damron F, Goldberg J (2012). Proteolytic regulation of alginate overproduction in *Pseudomonas aeruginosa*. Mol Microbiol.

[4] Maleki S, Almaas E, Zotchev SB, Valla S, Ertesvåg H (2016). Alginate biosynthesis factories in *Pseudomonas fluorescens*: localization and correlation with alginate production level. Appl Environ Microbiol.

[5] Bakkevig K, Sletta H, Gimmestad M, Aune R, Ertesvåg H, Degnes K, Christensen BE, Ellingsen TE, Valla S (2005). Role of the *Pseudomonas fluorescens* alginate lyase (AlgL) in clearing the periplasm of alginates not exported to the extracellular environment. J Bacteriol.

[6] Whitney JC, Whitfield GB, Marmont LS, Yip P, Neculai AM, Lobsanov YD, Robinson H, Ohman DE, Howell PL (2015). Dimeric c-di-GMP is required for post-translational regulation of alginate production in *Pseudomonas aeruginosa*. J Biol Chem.

[7] Hay ID, Remminghorst U, Rehm BH (2009). MucR, a novel membrane-associated regulator of alginate biosynthesis in *Pseudomonas aeruginosa*. Appl Environ Microbiol.

[8] Withers TR, Yin Y, Yu HD. Identification of novel genes associated with alginate production in *Pseudomonas aeruginosa* using mini-himar1 mariner transposon-mediated mutagenesis. J Vis Exp. 2014;85. 10.3791/51346 PMC4145724 24637508

[9] Ramos JL, Marques S, Timmis KN (1997). Transcriptional control of the Pseudomonas TOL plasmid catabolic operons is achieved through an interplay of host factors and plasmid-encoded regulators. Annu Rev Microbiol.

[10] Gimmestad M, Sletta H, Karunakaran P, Bakkevig K, Ertesvåg H, Ellingsen TE, Skjåk-Bræk G, Valla S (2002). New mutant strains of *Pseudomonas fluorescens* and variants thereof, methods of their production, and uses thereof in alginate production. WO2004/011628.

[11] Nishijyo T, Haas D, Itoh Y (2001). The CbrA-CbrB two-component regulatory system controls the utilization of multiple carbon and nitrogen sources in *Pseudomonas aeruginosa*. Mol Microbiol.

[12] Zhang XX, Rainey PB (2008). Dual involvement of CbrAB and NtrBC in the regulation of histidine utilization in *Pseudomonas fluorescens* SBW25. Genetics.

[13] Sonnleitner E, Valentini M, Wenner N, Haichar FZ, Haas D, Lapouge K (2012). Novel targets of the CbrAB/Crc carbon catabolite control system revealed by transcript abundance in *Pseudomonas aeruginosa*. PLoS One.

[14] Amador CI, Canosa I, Govantes F, Santero E (2010). Lack of CbrB in *Pseudomonas putida* affects not only amino acids metabolism but also different stress responses and biofilm development. Environ Microbiol.

[15] Hervás AB, Canosa I, Little R, Dixon R, Santero E. NtrC-dependent regulatory network for nitrogen assimilation in *Pseudomonas putida*. J Bacteriol. 2009;191(19):6123–35. 10.1128/JB.00744-09 PMC2747892 19648236

[16] Rehm N, Buchinger S, Strösser J, Dotzauer A, Walter B, Hans S, Bathe B, Schomburg D, Krämer R, Burkovski A (2010). Impact of adenylyltransferase GlnE on nitrogen starvation response in *Corynebacterium glutamicum*. J Biotechnol.

[17] Lee CR, Park YH, Kim M, Kim YR, Park S, Peterkofsky A, Seok YJ. Reciprocal regulation of the autophosphorylation of enzyme I^Ntr^ by glutamine and alpha-ketoglutarate in *Escherichia coli*. Mol Microbiol. 2013;88(3):473–85. 10.1111/mmi.12196 PMC3633653 23517463

[18] Pflüger-Grau K, de Lorenzo V (2014). From the phosphoenolpyruvate phosphotransferase system to selfish metabolism: a story retraced in *Pseudomonas putida*. FEMS Microbiol Lett.

[19] Lessie TG, Phibbs PV (1984). Alternative pathways of carbohydrate utilization in pseudomonads. Annu Rev Microbiol.

[20] Velazquez F, Pfluger K, Cases I, De Eugenio LI, de Lorenzo V (2007). The phosphotransferase system formed by PtsP, PtsO, and PtsN proteins controls production of polyhydroxyalkanoates in *Pseudomonas putida*. J Bacteriol.

[21] Cabeen MT, Leiman SA, Losick R. Colony-morphology screening uncovers a role for the *Pseudomonas aeruginosa* nitrogen-related phosphotransferase system in biofilm formation. Mol Microbiol. 2016;99(3):557–70. 10.1111/mmi.13250 PMC5130288 26483285

[22] Lamarche MG, Wanner BL, Crépin S, Harel J (2008). The phosphate regulon and bacterial virulence: a regulatory network connecting phosphate homeostasis and pathogenesis. FEMS Microbiol Rev.

[23] Marzan LW, Shimizu K (2011). Metabolic regulation of *Escherichia coli* and its *phoB* and *phoR* genes knockout mutants under phosphate and nitrogen limitations as well as at acidic condition. Microb Cell Fact.

[24] Kim HY, Schlictman D, Shankar S, Xie Z, Chakrabarty AM, Kornberg A (1998). Alginate, inorganic polyphosphate, GTP and ppGpp synthesis co-regulated in *Pseudomonas aeruginosa*: implications for stationary phase survival and synthesis of RNA/DNA precursors. Mol Microbiol.

[25] Dove SL, Hochschild A. Bacterial two-hybrid analysis of interactions between region 4 of the σ^70^ subunit of RNA polymerase and the transcriptional regulators Rsd from *Escherichia coli *and AlgQ from *Pseudomonas aeruginosa*. J Bacteriol. 2001;183(21):6413–21. 10.1128/JB.183.21.6413-6421.2001 PMC100137 11591686

[26] Marqués S, Manzanera M, González-Pérez MM, Gallegos MT, Ramos J. The XylS-dependent *Pm* promoter is transcribed in vivo by RNA polymerase with σ^32^ or σ^38^ depending on the growth phase. Mol Microbiol. 1999;31(4):1105–13. 10.1046/j.1365-2958.1999.01249.x 10096078

[27] Gisin J, Schneider A, Nagele B, Borisova M, Mayer C (2013). A cell wall recycling shortcut that bypasses peptidoglycan de novo biosynthesis. Nat Chem Biol.

[28] Yoshioka S, Newell PD (2016). Disruption of de novo purine biosynthesis in *Pseudomonas fluorescens* Pf0-1 leads to reduced biofilm formation and a reduction in cell size of surface-attached but not planktonic cells. PeerJ.

[29] Hamilton S, Bongaerts RJ, Mulholland F, Cochrane B, Porter J, Lucchini S, Lappin-Scott HM, Hinton JC (2009). The transcriptional programme of *Salmonella enterica* serovar Typhimurium reveals a key role for tryptophan metabolism in biofilms. BMC Genomics.

[30] Wood LF, Leech AJ, Ohman DE (2006). Cell wall-inhibitory antibiotics activate the alginate biosynthesis operon in *Pseudomonas aeruginosa*: Roles of sigma (AlgT) and the AlgW and Prc proteases. Mol Microbiol.

[31] Reiling SA, Jansen JA, Henley BJ, Singh S, Chattin C, Chandler M, Rowen DW (2005). Prc protease promotes mucoidy in *mucA* mutants of *Pseudomonas aeruginosa*. Microbiology.

[32] Damron FH, Barbier M, McKenney ES, Schurr MJ, Goldberg JB (2013). Genes required for and effects of alginate overproduction induced by growth of *Pseudomonas aeruginos*a on Pseudomonas isolation agar supplemented with ammonium metavanadate. J Bacteriol.

[33] Gimmestad M, Sletta H, Ertesvåg H, Bakkevig K, Jain S, Suh S-j, Skjåk-Bræk G, Ellingsen TE, Ohman DE, Valla S (2003). The *Pseudomonas fluorescens* AlgG protein, but not its mannuronan C5-epimerase activity, is needed for alginate polymer formation. J Bacteriol.

[34] Correa E, Sletta H, Ellis DI, Hoel S, Ertesvåg H, Ellingsen TE, Valla S, Goodacre R (2012). Rapid reagentless quantification of alginate biosynthesis in *Pseudomonas fluorescens* bacteria mutants using FT-IR spectroscopy coupled to multivariate partial least squares regression. Anal Bioanal Chem.

[35] Østgaard K (1992). Enzymatic microassay for the determination and characterization of alginates. Carbohydr Polym.

[36] de Lorenzo V, Cases I, Herrero M, Timmis KN (1993). Early and late response of TOL promoters to pathway inducers: Identification of postexponential promoters in *Pseudomonas putida* with *lacZ-tet* bicistronic reporters. J Bacteriol.

[37] Rainey PB, Bailey MJ (1996). Physical and genetic map of the *Pseudomonas fluorescens* SBW25 chromosome. Mol Microbiol.

