# Supplementary material for: Identification of genes affecting alginate biosynthesis in Pseudomonas fluorescens by screening a transposon insertion library
Source: BMC Genomics. 2017 Jan 3;18:11. doi: 10.1186/s12864-016-3467-7 (PMC5210274; doi:10.1186/s12864-016-3467-7)
Supplement: Additional file 1: Figure S1. — Growth profiles of Pseudomonas fluorescence SBW25 and MS2 cultivated in 0.5 x PIA. (PPTX 75 kb) [file 12864_2016_3467_MOESM1_ESM.pptx]

## Slide 1
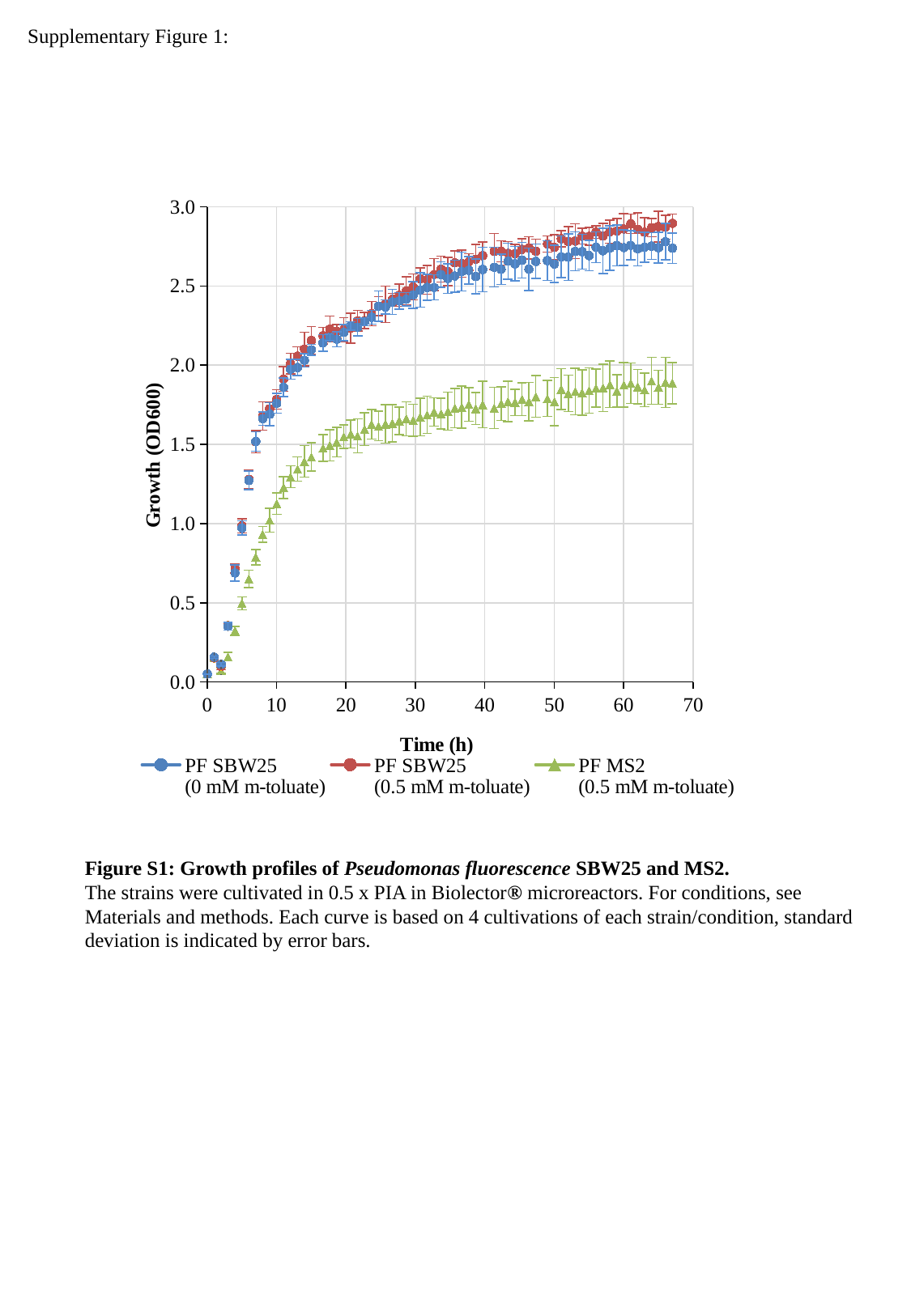

Supplementary Figure 1:
### Chart
| Category | PF SBW25
(0 mM m-toluate) | PF SBW25
(0.5 mM m-toluate) | PF MS2
(0.5 mM m-toluate) |
|---|---|---|---|Figure S1: Growth profiles of Pseudomonas fluorescence SBW25 and MS2.
The strains were cultivated in 0.5 x PIA in Biolector® microreactors. For conditions, see Materials and methods. Each curve is based on 4 cultivations of each strain/condition, standard deviation is indicated by error bars.
